# Thermoresponsive Smart Copolymer Coatings Based on P(NIPAM-*co*-HEMA) and P(OEGMA-*co*-HEMA) Brushes for Regenerative Medicine

**DOI:** 10.1021/acsbiomaterials.3c00917

**Published:** 2023-10-24

**Authors:** Svitlana Tymetska, Yana Shymborska, Yurij Stetsyshyn, Andrzej Budkowski, Andrzej Bernasik, Kamil Awsiuk, Volodymyr Donchak, Joanna Raczkowska

**Affiliations:** † Jagiellonian University, Doctoral School of Exact and Natural Sciences, Łojasiewicza 11, 30-348 Kraków, Poland; ‡ Jagiellonian University, Faculty of Physics, Astronomy and Applied Computer Science, Smoluchowski Institute of Physics, Łojasiewicza 11, 30-348 Kraków, Poland; § Lviv Polytechnic National University, St. George’s Square 2, 79013 Lviv, Ukraine; ∥ Faculty of Physics and Applied Computer Science, AGH - University of Science and Technology, al. Mickiewicza 30, 30-049 Kraków, Poland

**Keywords:** smart polymer brushes, thermoresponsive polymer, LCST, UCST, cell sheet engineering

## Abstract

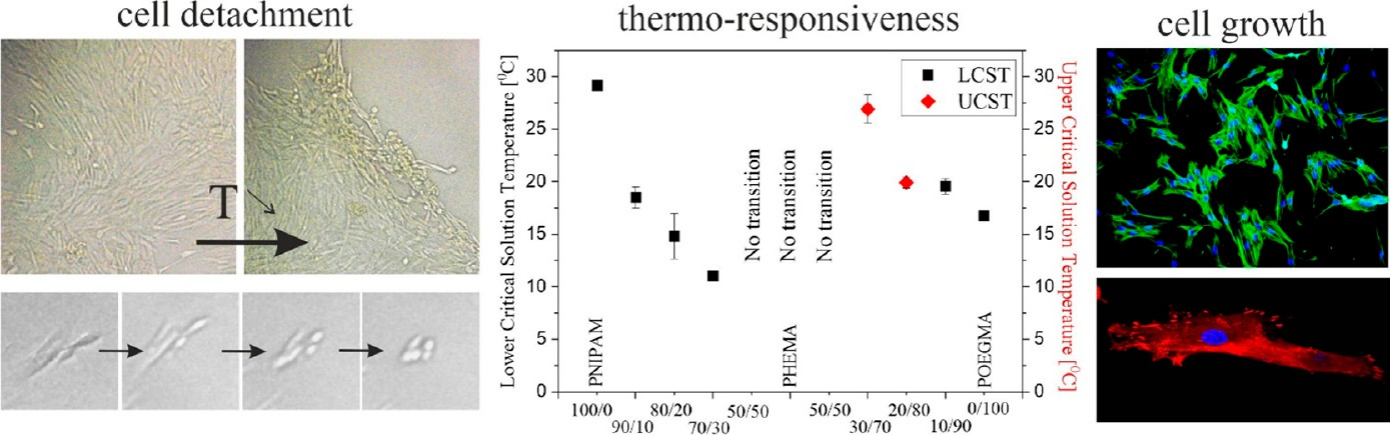

The fabrication of multifunctional, thermoresponsive platforms for regenerative medicine based on polymers that can be easily functionalized is one of the most important challenges in modern biomaterials science. In this study, we utilized atom transfer radical polymerization (ATRP) to produce two series of novel smart copolymer brush coatings. These coatings were based on copolymerizing 2-hydroxyethyl methacrylate (HEMA) with either oligo(ethylene glycol) methyl ether methacrylate (OEGMA) or *N*-isopropylacrylamide (NIPAM). The chemical compositions of the resulting brush coatings, namely, poly(oligo(ethylene glycol) methyl ether methacrylate-*co*-2-hydroxyethyl methacrylate) (P(OEGMA-*co*-HEMA)) and poly(*N*-isopropylacrylamide-*co*-2-hydroxyethyl methacrylate) (P(NIPAM-*co*-HEMA)), were predicted using reactive ratios of the monomers. These predictions were then verified using time-of-flight-secondary ion mass spectrometry (ToF-SIMS) and X-ray photoelectron spectroscopy (XPS). The thermoresponsiveness of the coatings was examined through water contact angle (CA) measurements at different temperatures, revealing a transition driven by lower critical solution temperature (LCST) or upper critical solution temperature (UCST) or a vanishing transition. The type of transition observed depended on the chemical composition of the coatings. Furthermore, it was demonstrated that the transition temperature of the coatings could be easily adjusted by modifying their composition. The topography of the coatings was characterized using atomic force microscopy (AFM). To assess the biocompatibility of the coatings, dermal fibroblast cultures were employed, and the results indicated that none of the coatings exhibited cytotoxicity. However, the shape and arrangement of the cells were significantly influenced by the chemical structure of the coating. Additionally, the viability of the cells was correlated with the wettability and roughness of the coatings, which determined the initial adhesion of the cells. Lastly, the temperature-induced changes in the properties of the fabricated copolymer coatings effectively controlled cell morphology, adhesion, and spontaneous detachment in a noninvasive, enzyme-free manner that was confirmed using optical microscopy.

## 1 Introduction

Regenerative medicine focuses on healing or replacing damaged tissues or organs, including dermal wounds, bone injuries, cardiovascular diseases, different types of cancer, and more.^1^ The traditional therapy based on transplantation of intact organs or tissues suffers from a very limited donor supply, and the need for new approaches, offering the potential for regenerating various tissues and organs of the human body, arose significantly in the last few decades.^2^ Proposed approaches include tissue engineering, which aims to restore, maintain, or improve tissue functions that are defective or have been lost by different pathological conditions, either by developing biological substitutes or by reconstructing tissues, and relies on the use of scaffolds providing the appropriate environment for the growth and proliferation of cells, leading to tissue regeneration.^3^ Therefore, biomaterials acting as synthetic frameworks for cell culture play a critical role in this technology, and a lot of effort is put into the development of artificial materials allowing spatiotemporal control of cell–matrix interactions. Regardless of the tissue type, a number of key factors are important when considering the biomaterial, including biocompatibility, biodegradability, mechanical properties, architecture, and manufacturing technology.^2,4^

In general, biomaterials can be divided into five main groups: natural and synthetic polymers, metals, ceramics, hybrid materials, and decellularized tissues.^5−7^ These materials are designed to help the reconstitution of functional tissues with increasing complexity (e.g., cocultures of several cell types, multilayered cell sheets, organoids, up to the “grail” of a whole organ).^8^

Presently, emphasis is placed on the design of polymeric scaffolds due to their advantages over other materials. Polymers have synthetic flexibility with a variety of functional groups. They show excellent mechanical and chemical robustness; they can be easily patterned, and many of them are nontoxic to cells.^6^

Especially, hydrogels, highly hydrated cross-linked polymer networks, have emerged as powerful synthetic analogues of extracellular matrices for basic cell studies as well as promising biomaterials for regenerative medicine applications.^9,10^ A critical advantage of these artificial matrices over natural networks is that bioactive functionalities, such as cell adhesive sequences and growth factors, can be incorporated in precise densities while the substrate mechanical properties are independently controlled.^11,12^ Among the most significant synthetic hydrogels are poly(2-hydroxethyl methacrylate) (PHEMA), polyethylene glycol (PEG), and poly(vinyl alcohol) (PVA).^7^ PHEMA finds an increasing number of applications in various biomedical fields, as it is easy to polymerize, not toxic, and highly resistant to degradation and possesses chemical groups that can be used for further modification of the coating.^13^ However, PHEMA itself does not provide sufficient conditions for cell culture. PHEMA-coated culture dishes decrease the adhesiveness and alter the shape of cells, which tend to form agglomerates of round cells. To enable the growth and proliferation of cells, chemical or biological modification of PHEMA is required.^14^

Although conventional tissue engineering based on the use of scaffolds is widely applied and enables the successful treatment of numerous deficiencies, it also has some drawbacks. First of all, there is a high rate of cell death or loss due to various factors, such as graft site inflammation, autoimmunity, or mechanical injury.^15^ Second, the free space created during prolonged degradation of the scaffold is filled with proliferated cells and extracellular matrix (ECM) proteins, such as collagen, which frequently leads to fibrosis, which is a pathological state.^16^ Third, the conventional harvesting of cells from the scaffold by trypsin digestion damages cell–cell interactions, cell–ECM interactions, and cell membrane proteins, resulting in decreased cell adhesion and proliferation and disrupting the newly formed tissue.^17,18^ To overcome these limitations, a new technique called “cell sheet technology” (CSE) was proposed by the group of Okano.^16,19,20^ In this technique, cell sheets are prepared using culture dishes modified with temperature-responsive polymers, which allow different cell types to adhere and proliferate at 37 °C and induce their spontaneous detachment for lowered temperature.^16−19,21−26^ Since CSE maintains the intact cell matrix, it provides an excellent microenvironment for vascularization and formation of complex tissues for regeneration of bones, heart, liver, muscles, cornea, periodontium, and other organs.^15,16,21,24,27^

Spontaneous detachment of cells from the scaffold is possible due to the unique properties of stimuli-responsive “smart” polymers, which are able to change physicochemical properties upon external factors, such as temperature, pH, light, etc.^28−30^ In recent years, responsive “smart” nanocoatings have been widely used as “controllable materials”, e.g., for liquid crystal orientation,^31,32^ generation of protein gradients,^33^ switching between superoleophobicity and superoleophilicity,^34^ or wettability by an ionic liquid.^35^ Polymer nanocoatings often include grafted polymer brushes.^36^ Among the diversities of responsive polymers, temperature-responsive polymers deserve special attention.^37−41^

Temperature-responsive systems can be synthesized in different forms, for example, macromolecules, gels, micelles, capsules, cross-linked films, grafted brushes, etc. The grafted polymer brushes are the most prospective for biomedical applications.^42−45^ At high grafting densities, i.e., when the distance between neighboring grafting points is small, steric repulsion leads to chain stretching and a brush-type conformation of the surface-tethered chains.^46^ At lower grafting densities, surface-tethered polymer chains can adopt various other conformations, which are referred to as mushroom or pancake.^47^ In our former works,^48−54^ a fabrication method of temperature-responsive coatings using graft polymerization “from the surface” of oligoperoxide or atom transfer radical polymerization (ATRP) initiator, grafted to a native glass surface functionalized with (3-aminopropyl)triethoxysilane (APTES), was developed. In particular, there is an increased interest in surfaces modified with temperature-responsive grafted polymer brushes, which can change their affinity toward proteins and cells under external stimuli and therefore have potential applications in biology and medicine.

Poly(*N*-isopropylacrylamide) (PNIPAM) is the most extensively studied thermoresponsive polymer for therapeutic purposes. It has a lower critical solution temperature (LCST) value at approximately 32 °C, is soluble at room temperature, and phase separates at body temperature (37 °C).^55−58^ Due to its unique physical and chemical properties, it has many applications, such as biosensors, tissue engineering, and drug delivery.^59−61^ Cells stick to dehydrated PNIPAM films as well as hydrogels (37 °C), while they cannot adhere to hydrated PNIPAM films (20 °C). Although PNIPAM is one of the most frequently used materials for CSE, other temperature-responsive polymer brushes, mainly based on poly(2-(2-methoxyethoxy)ethyl methacrylate) (POEGMA) or poly(2-substitued-2-ooxazoline)s (POx) are also considered as materials for CSE. However, they suffer from a very narrow range of brush thickness where spontaneous detachment of cells is possible.^62^ Temperature-induced cell detachment was also reported for upper critical solution temperature (UCST)-based materials composed of poly(*N*-acryloyl glycinamide-*co*-*N*-phenylacrylamide) copolymers.^63^

Despite the great advances in the usage of polymers for tissue engineering, this field still has enormous research potential in both application and basic research. The main challenges include minimizing undesirable side effects, maximizing cellular viability and the ability to form new tissue, reducing “detachment” time, preventing culture inflammation, and broadening the range of cell types that can be cultured. On the other hand, also some basic questions about the mechanisms driving cellular behavior on substrates with given physicochemical properties, including polymer assembly, surface topography or chemical cues, nano- or macrostructure, biocompatibility, biodegradability, mechanical properties, directing cell function, and induced formation of natural tissue, remain unanswered. These issues may be resolved by the creation of completely new materials, as well as the synergistic implementation of the existing ones.

In this work, we used atom transfer radical polymerization (ATRP) to fabricate new smart polymer brush coatings based on two copolymers that include hydroxilic groups, namely, poly(oligo(ethylene glycol) methyl ether methacrylate-*co*-2-hydroxyethyl methacrylate) (P(OEGMA-*co*-HEMA)) and poly(*N*-isopropylacrylamide)-*co*-2-hydroxyethyl methacrylate (P(NIPAM-*co*-HEMA)) with different molar fractions. These coatings have at least two advantages in comparison to homopolymers: PNIPAM or POEGMA. First, they can be easily functionalized using the interaction of the hydroxylic groups of the HEMA with biologically active substances, for example, RGD peptides. Second, the mechanism and intensity of the temperature-induced response as well as the transition temperature can be easily modulated by changing the amount of HEMA in the coating or modifying components in the postpolymerization reactions. On the other hand, a critical challenge in copolymer coatings is the preservation of their thermoresponsive properties.

The chemical composition of prepared coatings was verified using time-of-flight-secondary ion mass spectrometry (ToF-SIMS) and X-ray photoelectron spectroscopy (XPS), whereas their thermoresponsiveness was examined using water contact angle (CA) measurements at different temperatures. In turn, the topography of the coatings was traced by using atomic force microscopy (AFM).

Then, the possibility of the application of the fabricated coatings as materials for CSE platforms was verified. For this purpose, first, the noncytotoxic character of the coatings was confirmed by a detailed examination of cell growth, visualized using optical and fluorescence microscopy, and cell viability, examined using MTT colorimetric tests and live/dead staining. Then, the formation of adhesive focal sites in the early adhesion stages was visualized by vinculin staining. Finally, the possibility to control the morphology, adhesion, and detachment of cells and the impact of incubation in temperature lowered below the transition temperature on the morphology of cells were verified for all types of examined coatings using optical microscopy.

## 2 Experimental Section

### 2.1 Grafting of the Brushes

The brushes of POEGMA, PNIPAM, PHEMA, P(OEGMA-*co*-HEMA), and P(NIPAM-*co*-HEMA) were grafted to 15 × 15 mm glass plates. The ATRP requires three steps: (I) activation of the glass surface by (3-aminopropyl) triethoxysilane (APTES), (II) grafting ATRP initiator 2-bromoisobutyryl bromide (BiBB) to amino groups of the activated glass surface by APTES, and (III) polymerization of the brushes on glass plates modified with (BiBB).

To activate the glass surface with APTES, it was previously washed three times in an ultrasonic bath (Emmi-12HC, EMAG, Germany) in ethanol and treated with plasma cleaner (Zepto, Diener electronic, Germany). After, clean glass plates were immersed for 10 min in the 2% (w/w) solution of APTES in toluene. After the glass plates were removed, they were washed three times with an ultrasonic bath in toluene and dried in the hot plate at 120 °C for 30–40 min. Finally, the glass plates were remined for 5 min at ambient temperature and were ready for further modification.

To graft the ATRP initiator, 1.3 mL of BiBB and 1.5 mL of triethylamine were mixed in 50 mL of tetrahydrofuran, and the activated glass plates by APTES were immersed for 40 min. Then, the glass plates were removed and washed three times with an ultrasonic bath in dichloromethane and dried by a nitrogen stream.

Finally, to graft the brushes, the monomers were dissolved in a solution of 16 mL of methanol and 4 mL of water and bubbled with nitrogen in a Schlenk flask for 10–12 min. The oligo(ethylene glycol) methyl ether methacrylate (OEGMA) was previously passed through the Al_2_O_3_ column. Then, 14 mg of CuBr_2_ and 51.5 mg of sodium l-ascorbate were added to the solution and bubbled with nitrogen for the next 10–12 min. The concentrations of the monomers are listed in Table 1. Subsequently, the glass plates with grafted initiator were immersed into the solution for 12 h overnight. Finally, the glass plates were removed and washed three times with an ultrasonic bath in methanol and dried with nitrogen stream. After this, the polymer brushes were ready.

**Table 1:** Concentration of Monomers

brush	concentrations of monomers
POEGMA	OEGMA: 11.2800 g
P(OEGMA-*co*-HEMA) 50/50	OEGMA: 5.3580 g; HEMA: 4.0950 g
P(OEGMA-*co*-HEMA) 70/30	OEGMA: 7.5576 g; HEMA: 2.5740 g
P(OEGMA-*co*-HEMA) 80/20	OEGMA: 8.7420 g; HEMA: 1.7550 g
P(OEGMA-*co*-HEMA) 90/10	OEGMA: 10.1520 g; HEMA: 0.7800 g
PNIPAM	NIPAM: 6.7800 g
P(NIPAM-*co*-HEMA) 50/50	NIPAM: 6.1020 g; HEMA: 0.7800 g
P(NIPAM-*co*-HEMA) 70/30	NIPAM: 6.7574 g; HEMA: 0.0260 g
P(NIPAM-*co*-HEMA) 80/20	NIPAM: 6.7721 g; HEMA: 0.0091 g
P(NIPAM-*co*-HEMA) 90/10	NIPAM: 6.7766 g; HEMA: 0.0039 g
PHEMA	HEMA: 7.8000 g

### 2.2 XPS X-ray Photoelectron Spectroscopy (XPS)

The X-ray photoelectron spectroscopy measurements were performed with a PHI VersaProbe II apparatus. The samples were irradiated with a focused monochromatic Al Kα (*E* = 1486.6 eV) X-ray beam with a diameter of 100 μm, and the beam was rastered over an area of 400 × 400 μm^2^. The pass energy of the analyzer was set to 46.95 eV, and double neutralization with electrons and low energy monatomic Ar^+^ ions was used to avoid charging effects. Spectra were referenced to the neutral (C–C) carbon C 1s peak at a binding energy of 284.80 eV.

### 2.3 Time of Flight-Secondary Ion Mass Spectrometry (ToF-SIMS)

To examine the surface chemistry, ToF-SIMS was performed using the TOF.SIMS 5 instrument (ION-TOF GmbH), equipped with a 30 keV bismuth liquid metal ion gun. Bi_3_ clusters were used as primary ions with an ion dose density lower than 10^12^ ion/cm^2^ to ensure static mode conditions. A pulsed low-energy electron flood gun was used for charge compensation. For each sample, high mass resolution spectra of negative and positive ions were acquired from six different and nonoverlapping spots (200 μm × 200 μm area).

### 2.4 Atomic Force Microscopy

Topographic images were recorded on randomly chosen regions of the sample surface. Measurements were carried out in the air using the commercially available Agilent 5500 system (Keysight) working in noncontact mode with noncoated super sharp silicon probes. For every coating, at least three images were analyzed using the AFM apparatus.

### 2.5 Profilometry

To examine the thickness of the fabricated coatings, they were scratched, and the scratch profiles were recorded using a Dektak XT (Bruker, Germany) profilometer, equipped with a 12.5 μm radius stylus. For each sample, at least three profiles were collected in the standard hills and valleys module to determine the average thickness of the coatings.

### 2.6 Water Contact Angles

Static contact angle experiments were performed by the sessile drop technique using a Kruss EasyDrop (DSA15) instrument with a Peltier temperature-controlled chamber. A few (5–10) drops of the water were placed on the fabricated coatings and pictured with an LCD camera, and the water contact angles were measured by the software provided by the device producer. The water contact angles were measured at temperatures ranging from 6 to 32 °C (for POEGMA and P(OEGMA-*co*-HEMA)) and 6 to 42 °C (for PNIPAM and P(NIPAM-*co*-HEMA)) each 3 °C to record the thermoresponsiveness of the coatings after at least 10 min of stabilization in a given temperature. Contact angles were expressed as the average of the measurements at different spots.

### 2.7 Cell Culture

Human primary dermal fibroblasts Neonatal (HDFn; ATCC, PCS-201-010) were purchased from ATCC (Manassas, VA, USA). Cells were cultured in DMEM medium high glucose (Sigma-Aldrich, catalog number D6429), which was supplemented with a 10% fetal bovine serum (Sigma-Aldrich, F9665) and 1% penicillin–streptomycin–neomycin solution (Sigma-Aldrich, Darmstadt, Germany, P4083), in culture flasks at 37 °C in a humidified atmosphere in a CO_2_ incubator providing 95% air and 5% CO_2_. The glass coverslips 15 × 15 mm coated with polymer brushes were placed on the bottom of the cell culture plate (12-well; flat bottom). The samples were sterilized with 96% ethanol for 5 min, then rinsed 2 times with sterile, distilled water, and left in water for 2 h under a laminar flow chamber (Nu425, NuAire, Plymouth, MN, USA). After that, cells were placed over all types of coatings at a concentration of 5000 cells/cm^2^. Next, the cell culture plates were incubated in the CO_2_ incubator for 1, 3, or 7 days. The medium was replaced after 24 and 96 h of the study. For each experimental sequence, two or three identical samples were prepared and measured. All experiments were repeated at least three times in a time frame to prove the reproducibility of the results.

### 2.8 MTT Assay

The viability of cells was verified using an MTT calorimetric test (Cell Proliferation Kit I, Sigma-Aldrich, 11465007001). Briefly, fibroblasts were cultured in a multiwell plate (12 wells) in 1 mL of the corresponding culture medium. Next, 100 μL of MTT reagent (tetrazolium salt) was added to the cells in the culture medium. Cells were incubated at 37 °C in the incubator for 4 h. Then, 1 mL of the solubilization buffer was added to each well. The plate was left overnight in the incubator in a humidified atmosphere at +37 °C in 5% CO_2_. The MTT method is based on the reduction of the tetrazolium compound by viable cells to generate a colored formazan product that is soluble in a cell culture medium. The resulting colored solution was quantified by a scanning multiwell spectrophotometer (SPECTROstar Nano, BMG Labtech). The final volume of 2.1 mL was pipetted to a 24-well plate with 600 μL per hole. The absorbance was determined in the 24-well for each time frame of 1, 3, or 7 days at OD = 560 nm. The MTT assay was repeated at least three times at each time point.

### 2.9 Immunofluorescence Assay

For fluorescent staining of actin, vinculin, and the cell nuclei, the following protocol was applied. First, cells were fixed to the substrate by immersion in a solution of 3.7% paraformaldehyde in PBS (Thermo Scientific, 169650010) for 15 min at 37 °C. Later, cells were permeabilized with 0.1% Triton X-100 solution (Sigma, T8787) at room temperature for 8 min, and the samples were washed with PBS buffer for 2 min, blocked with 4% BSA for 1 h, and later incubated with primary antibody at a concentration of 5 μg/mL overnight at 4 °C (mouse monoclonal IgG antivinculin from Thermo Scientific, 14-9777-82). Subsequently, the cells were washed 3 times for 5 min with PBS buffer with 0.01% Tween 20. To dye the actin cytoskeleton, the cell nuclei, and vinculin, samples were incubated with a solution of Alexa Fluor 488 conjugated with phalloidin (Alexa Fluor 488 Phalloidin, Thermo Fisher Scientific, A12379) in 400× dilution, a 1 μg/mL solution of Hoechst 34580 dye (Thermo Fisher Scientific, H21486), and a secondary antibody at a concentration of 2 μg/mL (Alexa Fluor 633-conjugated goat antimouse IgG (Thermo Scientific, A-21050)) for 60 min. The cells were then thoroughly washed 2 times for 5 min with PBS buffer and 2 times for 5 min with water. Finally, stained samples were mounted on glass slides in DePex medium (Serva) and stored at 18 °C. The fluorescent images were collected using the Olympus IX51 microscope equipped with a 100 W Mercury light source (Olympus U-LH100HG), U-MWIG2 filter (λ_exit_ = 530–550 nm, λ_emit_ = 590 nm), and U-MNB2 filter (λ_exit_ = 470–490 nm, λ_emit_ = 520 nm). The fluorescent images of vinculin were taken under a ZEISS LSM 710 (release version 8.1) confocal microscope with a 40× oil immersion objective. For image processing, an ImageJ FIJI was used. For each experimental run, 10 fluorescent images from three substrates with stained cells were collected.

### 2.10 Live/Dead Staining

The differentiation of living and dead cells was conducted using a cell stain double staining kit (Sigma-Aldrich, 04511) for simultaneous fluorescence staining. This kit contains Calcein-AM and Propidium Iodide solutions, which allow for the differentiation of living (green fluorescence) and dead cells (red fluorescence). In a 7-day experiment, the medium was replaced by a fresh one 24 h after seeding the cells; in a 1-day experiment, the medium was not changed. 1 or 7 days after seeding, the cells were trypsinized, but all solutions used (medium from above the cells, PBS used for washing, and trypsin with detached cells) were poured into Eppendorf-type tubes to collect all cells. The suspension was centrifuged (300 rcf, 5 min), and the pellet was resuspended in 100 μL of fresh medium. The cell suspension was then mixed on a glass slide at a volume ratio of 1:1 with a staining kit solution. Live and dead cells were counted under an inverted Olympus IX51 fluorescence microscope.

## 3 Results and Discussion

### 3.1 Composition of the Coatings

Fabrication of the copolymer coating with a given molar composition requires very careful adjustment of the synthesis components, as the reactive ratios of monomers may differ significantly, resulting in the molar fractions in the synthesized coating being far from the weight fractions of monomers used in the process. OEGMA used in our work has a chemical structure similar to that described in ref (64), where the copolymerization process of HEMA with OEGMA was reported. For the statistical P(OEGMA-*co*-HEMA) copolymers, the calculated reactivity ratios were equal to *r*_OEGMA_ = 1.18 and *r*_HEMA_ = 0.95, suggesting that OEGMA is slightly more reactive than HEMA and that the distribution of the units in the polymer chain is practically random. On the contrary, for copolymerization of *N*-isopropylacrylamide (NIPAM) with HEMA, the calculated reactivity ratios differ significantly and are equal to *r*_NIPAM_ = 0.0034 and *r*_HEMA_ = 0.114.^65^ These values suggest a very clear-cut alternating behavior of the NIPAM monomer toward the HEMA monomer and HEMA random behavior toward NIPAM. Thus, the copolymer sequence probably consisted of a large sequence of alternating repeating units with some randomness, especially when the content of HEMA was larger in the copolymer.

The molar composition of the P(OEGMA-*co*-HEMA) and P(NIPAM-*co*-HEMA) coatings (Table 2) can be controlled by changing the ratio of the monomers in the reaction mixture and calculated according to eqs 1 and 2:^65^
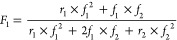
1
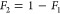
2where *F*_1_ and *F*_2_ are mole fractions of HEMA and OEGMA or NIPAM in the copolymer, respectively; *r* is the reactivity ratio for OEGMA/HEMA, *r*_1_ = 1.18 and *r*_2_ = 0.95, and for NIPAM/HEMA, *r*_1_ = 0.0034 and *r*_2_ = 0.114; *f*_2_ and *f*_2_ are the concentrations of HEMA and OEGMA or HEMA and NIPAM in the reaction mixture, respectively. Although the eqs 1 and 2 are mainly used for the instantaneous composition of two monomers in copolymers at low conversion rates (<15%), they can also be used to estimate the general tendencies in the structure of the grafted brushes.

**Table 2:** Calculated Composition of the Grafted Copolymer Brushes

		mole fractions of the segments in copolymer brush coatings	
sample	monomer molar ratios in the reaction mixture (OEGMA or NIPAM) to HEMA	HEMA	OEGMA or NIPAM
P(OEGMA-*co*-HEMA) 50/50	0.0285/0.0315	0.50	0.50
P(OEGMA-*co*-HEMA) 70/30	0.04020/0.01980	0.30	0.70
P(OEGMA-*co*-HEMA) 80/20	0.04650/0.01350	0.20	0.80
P(OEGMA-*co*-HEMA) 90/10	0.05400/0.00600	0.09	0.91
P(NIPAM-*co*-HEMA) 50/50	0.05400/0.00600	0.50	0.50
P(NIPAM-*co*-HEMA) 70/30	0.05980/0.00020	0.33	0.67
P(NIPAM-*co*-HEMA) 80/20	0.05993/0.00007	0.20	0.80
P(NIPAM-*co*-HEMA) 90/10	0.05997/0.00003	0.11	0.89

### 3.2 Physicochemical Properties of the Coating

The composition of the coatings was first determined using time-of-flight-secondary ion mass spectrometry (ToF-SIMS).

**Figure 1:**
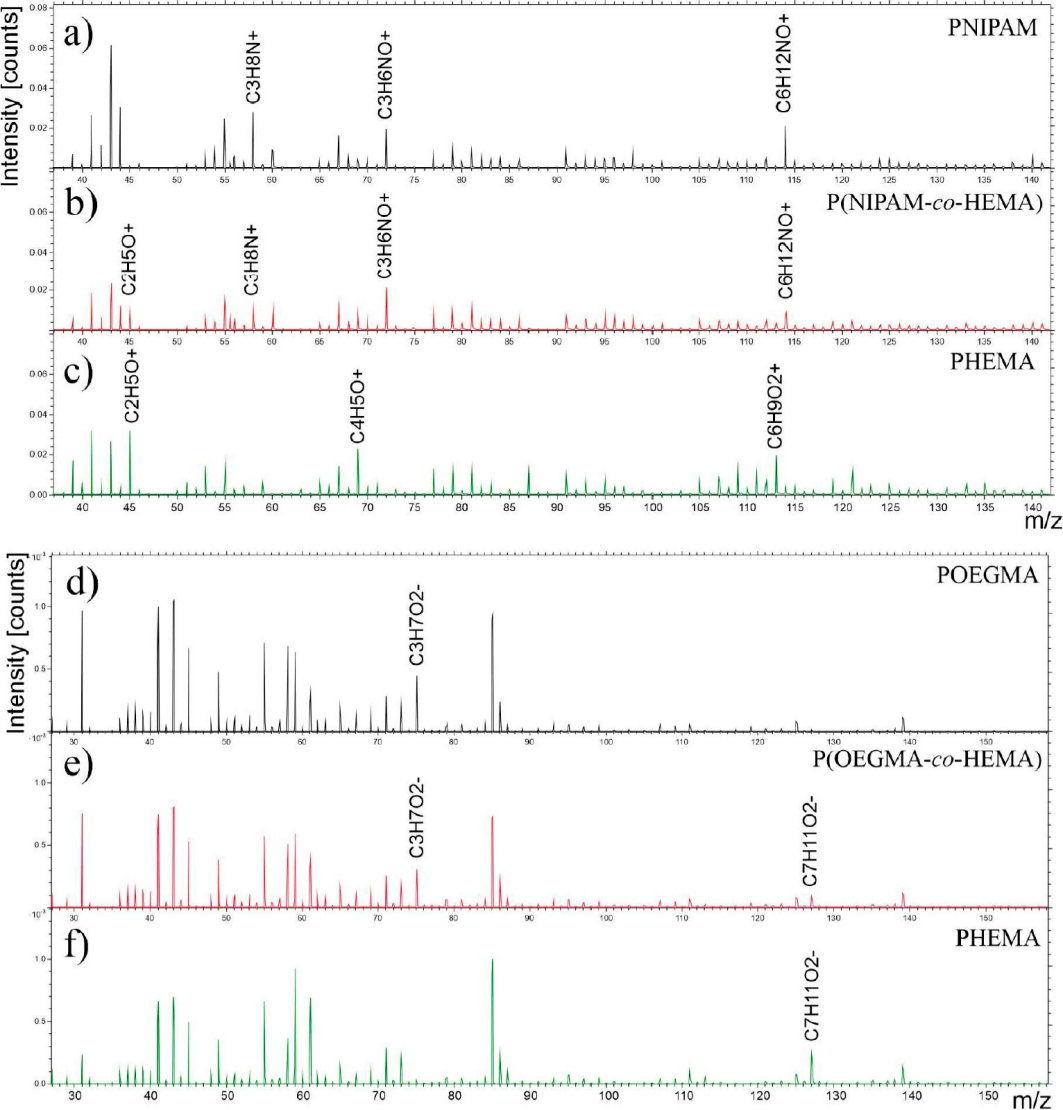
ToF-SIMS positive (a–c) and negative (d–f) ion spectra of PNIPAM (a), P(NIPM-*co*-HEMA) (b), PHEMA (c), POEGMA (d), and P(OEGMA-*co*-HEMA) (e), and PHEMA (f) polymer brushes synthesized using the ATRP method.

The positive ion spectra recorded for the PNIPAM coating (Figure 1a) show a series of peaks for *m*/*z* values equal to 58, 72, and 114, which correspond to positive C_3_H_8_N^+^, C_3_H_6_NO^+^, and C_6_H_12_NO^+^ ions characteristics for PNIPAM,^66^ whereas for spectra measured for the PHEMA brush (Figure 1c), a series of oxygen-containing hydrocarbons such as C_2_H_5_O^+^, C_4_H_5_O^+^, and C_6_H_9_O_2_^+^ ions, typical for PHEMA,^67^ may be observed. In turn, for the representative P(NIPAM-*co*-HEMA) copolymer brush (Figure 1b), peaks indicating the presence of both polymers, i.e., PNIPAM and PHEMA, are visible, thus confirming the successful fabrication of the coatings. In the case of the POEGMA brush (Figure 1d), to verify the successful fabrication of this coating, negative ion spectra were also recorded for POEGMA, PHEMA, and P(OEGMA-*co*-HEMA) brushes. The results presented in Figure 1d–f show the C_3_H_7_O_2_^–^ peak characteristic for POEGMA (Figure 1d), the C_7_H_11_O_2_^–^ signal representative for PHEMA (Figure 1f), and both mentioned peaks in the case of spectra recorded for copolymer brush (Figure 1e), confirming the proper synthesis of all coatings.

**Figure 2:**
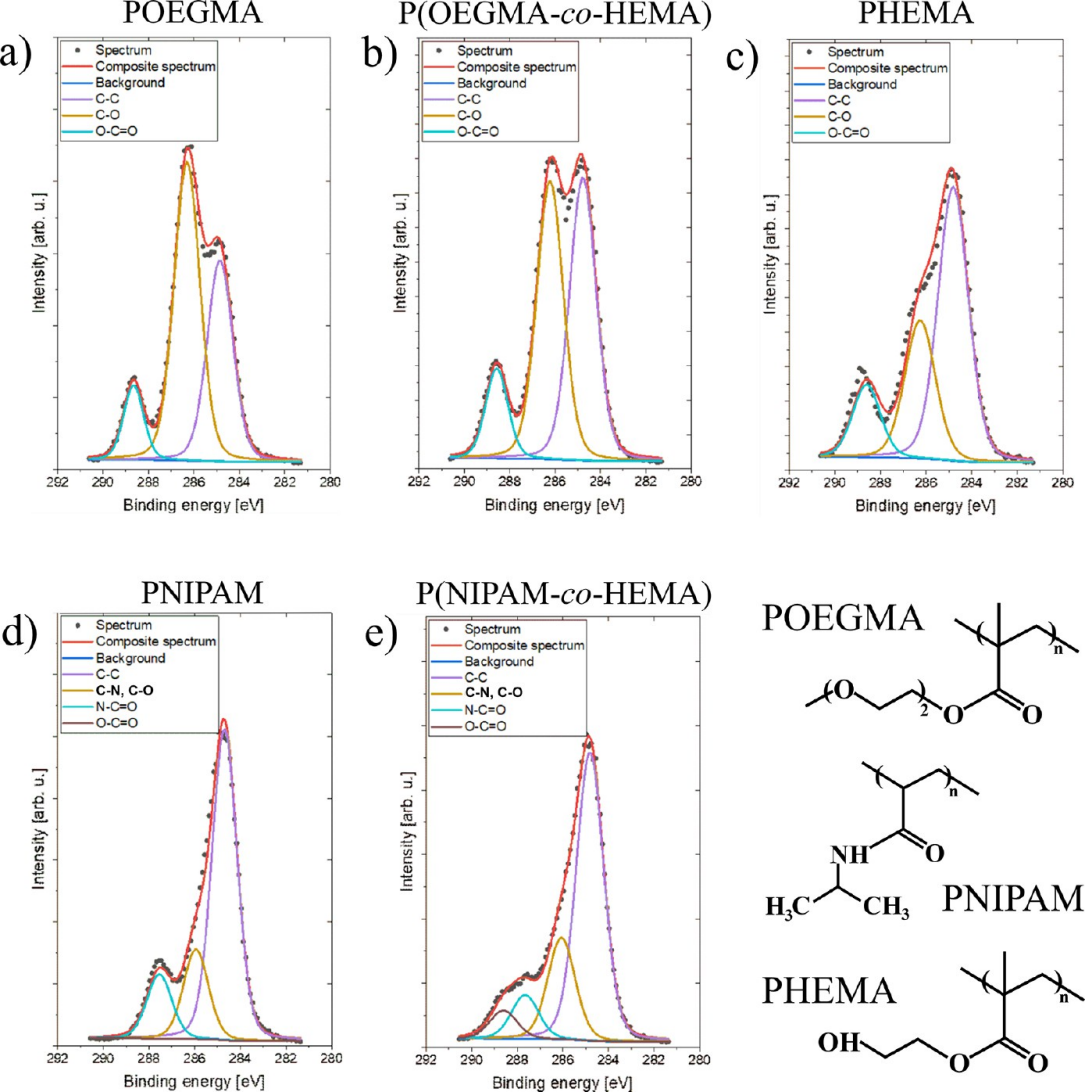
XPS C 1s core-level spectra of POEGMA (a), PHEMA (c), PNIPAM (d), and representative copolymer P(OEGMA-*co*-HEMA) (b) and P(NIPAM-*co*-HEMA) (e) coatings.

In addition to the ToF-SIMS measurements, the coatings were also examined by using the XPS technique, providing quantitative information about their chemical composition. First, the C 1s core-level XPS spectra were collected for coatings composed of pure polymers and their copolymers and resolved into a few contributions, characteristic for OEGMA, HEMA, or NIPAM (Figure 2).

The C 1s spectrum of the POEGMA brush (Figure 2a) consists of three peaks, corresponding to neutral carbon C–C (violet line, 284.8 eV) and two carbons with electron-efficient environments characteristic for OEGMA: C–O (orange line, 286.3 eV) and O–C=O (cyan line, 288.6 eV) bonds, with the lowest intensity for the O–C=O peak and that of C–O being the most intense. The spectrum recorded for the PHEMA coating (Figure 2c) is composed of the same sequence of peaks; however, the relation between their densities differs significantly, with the strongest signal corresponding to the C–C bond. In the case of the P(OEGMA-*co*-HEMA) coating (Figure 2b), the intensities of the C–O and C–C peaks are comparable, confirming the copolymer composition. In turn, the PNIPAM brush (Figure 2d) exhibits, in addition to the peak of the neutral carbon C–C (284.8 eV), also a contribution from the N–C=O (cyan line, 287.9 eV) bond, which is specific for NIPAM. Additionally, a strong peak at 286.3 eV, corresponding to the C–N bond, is observed. Finally, the spectra recorded for P(NIPAM-*co*-HEMA) coatings (Figure 2e) are composed of four peaks, corresponding to C–C, C–O (coincident with C–N), N–C=O, and −C=O bonds, as expected for a copolymer.

**Figure 3:**
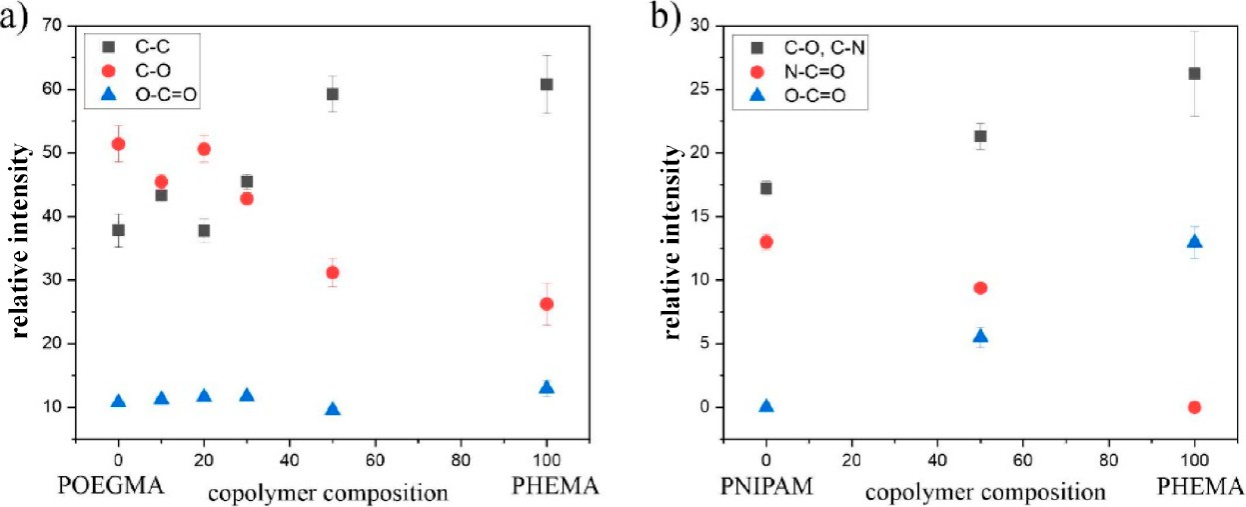
Relative intensities (percent) of peaks corresponding to C–C (black squares), C–O (red circles), and O–C=O (blue triangles) bonds in XPS C 1s core-level spectra of P(OEGMA-*co*-HEMA) coatings (a) and peaks corresponding to C–O, C–N (black squares), N–C=O (red circles), and O–C=O (blue triangles) bonds in XPS C 1s core-level spectra of P(NIPAM-*co*-HEMA) coatings (b).

Based on XPS data, the relative intensities of peaks characteristic of the examined polymers were determined (Figure 3). For the P(OEGMA-*co*-HEMA) coating (Figure 3a), a monotonic increase of C–O peak intensity, more characteristic for POEGMA, accompanied by a monotonic decrease of C–C peak intensity, more specific for PHEMA, may be observed for the increasing abundance of OEGMA in the coating. These results confirm the successful fabrication of polymer coatings with the predicted composition. In turn, for P(NIPAM-*co*-HEMA) brushes (Figure 3b), the intensity of the N–C=O peak related to PNIPAM increases, whereas the intensity of the O–C=O peak, characteristic for PHEMA, decreases with a higher content of PNIPAM in the coating. As shown in Section 3.1 and Table 2, the reactivity ratios for the pair of NIPAM and HEMA are significantly lower than 1 (*r*_1_ = 0.0034 and *r*_2_ = 0.114), and *r*_1_ is approximately 100 times smaller than *r*_2_, indicating a higher abundance of monomer sequences in such copolymers compared to a random copolymer. Additionally, these values suggest that NIPAM exhibits lower reactivity toward copolymerization compared to HEMA. In other words, HEMA is more likely to react with other monomers, resulting in a higher incorporation of HEMA units in the resulting copolymer structure. Analyzing this type of grafted brush coatings is challenging when one of the comonomers is present in low concentrations; therefore, we demonstrated exactly expressed information on HEMA units only for P(NIPAM-*co*-HEMA) brushes with a high calculated HEMA content. This limitation prevents a comprehensive analysis, as achieved with the P(OEGMA-*co*-HEMA) coatings. Nevertheless, even a small amount of HEMA units in P(NIPAM-*co*-HEMA) brushes significantly modifies the coating properties, allowing for precise tuning of the LCST in the region of the lowest values.

In the next step, the topography of the coatings was recorded using atomic force microscopy (AFM) depicting similar, island-like structures for all coatings (Figure S1). The numerical analysis of recorded topographies by means of root-mean-square (RMS) roughness analysis (Table S1) indicates that PNIPAM and POEGMA coatings are quite rough (∼2.5 nm) and the PHEMA coating is relatively smooth, with an RMS value less than half a nanometer, whereas the copolymer brushes have an intermediate roughness of about 1–1.5 nm.

**Figure 4:**
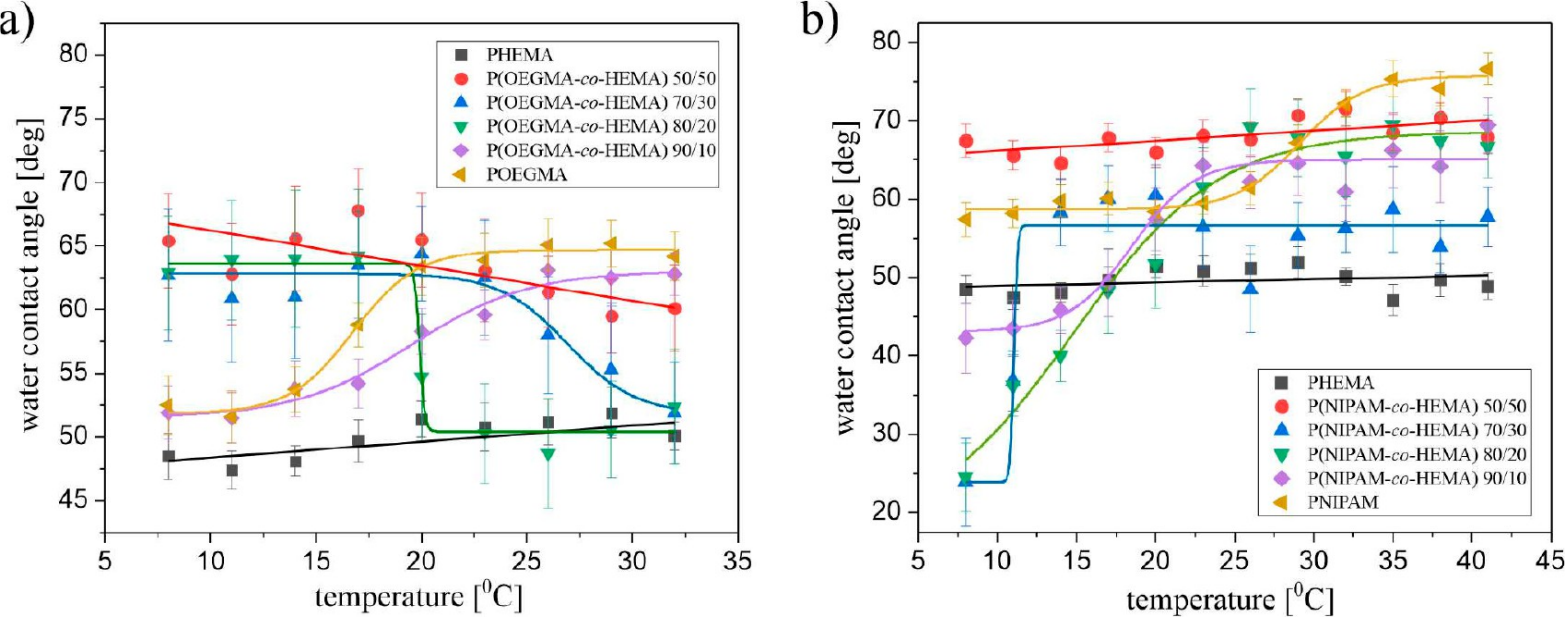
Plots of water contact angles as a function of the temperature of POEGMA, P(OEGMA-*co*-HEMA), and PHEMA (a) and PNIPAM, P(NIPAM-*co*-HEMA), and PHEMA (b).

In turn, to verify thermoresponsiveness of the coatings, their wettability was determined with water contact angle measurements (CA) as a function of temperature (Figure 4).

Recorded results show that both PNIPAM and POEGMA brushes (orange triangles in Figure 4a,b, respectively) undergo the temperature transition of the wettability, in accordance with our previous results.^48,68^ In the case of both polymers, the transition is driven by the lower critical solution temperature (LCST), with the transition temperature determined from the Boltzmann fit to the experimental points equal to 29 and 17 °C for PNIPAM and POEGMA, respectively. In contrast, the PHEMA coating does not show any significant transition in wettability, and the water CA remains equal to approximately 50° in the whole measured temperature range.

Analysis of the results depicts that the copolymerization of OEGMA with HEMA leads to the fabrication of coatings whose thermal response depends strongly on the composition. For P(OEGMA-*co*-HEMA) 50/50, the sensitivity of the coating to the temperature stimulus is very weak and no transition is noticed, whereas for polymer brushes with lower HEMA content, it is clearly visible. However, the mechanism and strength of the transition differ significantly. For the P(OEGMA-*co*-HEMA) 90/10 coating, with the very low fraction of HEMA, the LCST transition is observed, with the transition temperature shifted as compared to the POEGMA coating to 19.6 °C (see Table S2). In contrast, for copolymers containing higher amounts of HEMA, i.e., P(OEGMA-*co*-HEMA) 80/20 and 70/30, a well-pronounced UCST transition occurs. To explain the observed differences in the wettability response to temperature changes, we should consider the interactions between POEGMA, PHEMA, and water molecules. For POEGMA, the hydrogen bonds between the ether oxygens of OEGMA and water hydrogens are formed below the LCST and disrupted above the LCST when polymer–polymer interactions are favored. At a high content of HEMA in the copolymer (more than 50%), the copolymer, as well as PHEMA, becomes soluble over the entire investigated temperature range. Meanwhile, for P(OEGMA-*co*-HEMA) 80/20 and 70/30 samples that exhibit UCST, there is evidently a competitive formation of hydrogen bonds between the ether oxygens of OEGMA and the hydrogens of the hydroxyl groups in fragments of HEMA, which blocks the interaction with water molecules. Above UCST, these hydrogen bonds break, and the interaction of the hydroxyl groups of HEMA with water molecules is restored. At the same time, the OEGMA fragments remain relatively isolated and therefore cannot interact with each other, which means they do not exhibit LCST behavior. Also, transition temperature depends on the coating composition and decreases with the decreasing amount of HEMA. These results are in agreement with the literature data, which report the possibility of adjusting the transition temperature of P(OEGMA-*co*-HEMA) brushes in a wide range simply by changing the PHEMA content.^64^

Similarly, for P(NIPAM-*co*-HEMA) coatings, the temperature response of the coating depends on the composition. For the symmetric P(NIPAM-*co*-HEMA) 50/50 coating, no temperature transition is observed in the analyzed temperature range, and the water contact angle recorded for this coating equals 68°. However, for the P(NIPAM-*co*-HEMA) coatings with a lower content of HEMA, the LCST transition is visible.

**Figure 5:**
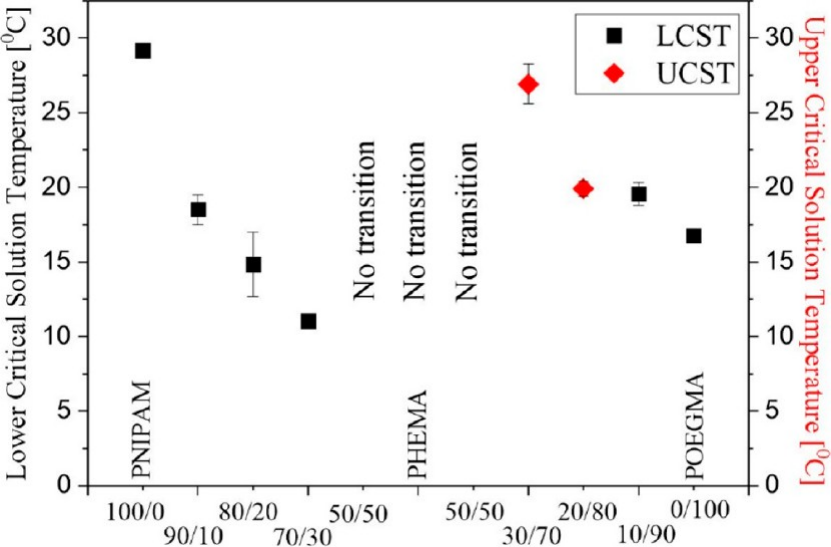
Transition temperature of the coatings.

Moreover, the changes in coating composition also lead to a modification of the transition temperature (Figure 5 and Table S2). For P(OEGMA-*co*-HEMA) coatings, the calculated temperature of transition decreases with a decreasing amount of HEMA, and this relation is not affected by the mechanism of transition. These results are in agreement with the literature data, which report the possibility of adjusting the transition temperature of P(OEGMA-*co*-HEMA) brushes in a wide range simply by changing the PHEMA content.^64^

In contrast, for P(NIPAM-*co*-HEMA) coatings, the opposite effect is observed, and the transition temperature increases with decreasing fractions of HEMA in the mixture. Similar changes in the LCST of P(NIPAM-*co*-HEMA) were observed for polymers synthesized by both the ultrasonic polymerization method^69,70^ and general radical polymerization.^71^ Such an effect was attributed to the increment of hydrophobicity due to the hydrogen-bonding between the hydroxyl groups in HEMA and the amide groups in NIPAM.^72,73^

### 3.3 Cytotoxicity of the Coatings

To assess the information about the potential cytotoxicity of the fabricated coatings, they were used as substrates for dermal fibroblasts culture. To exclude the influence of the specific behavior of the thermoresponsive coatings at different temperatures, we determined the cytotoxicity for the symmetric coatings of both types, where a thermal transition was not observed. The growth, morphology, and viability of cells were examined to provide information about the impact of the coatings on human cells.

**Figure 6:**
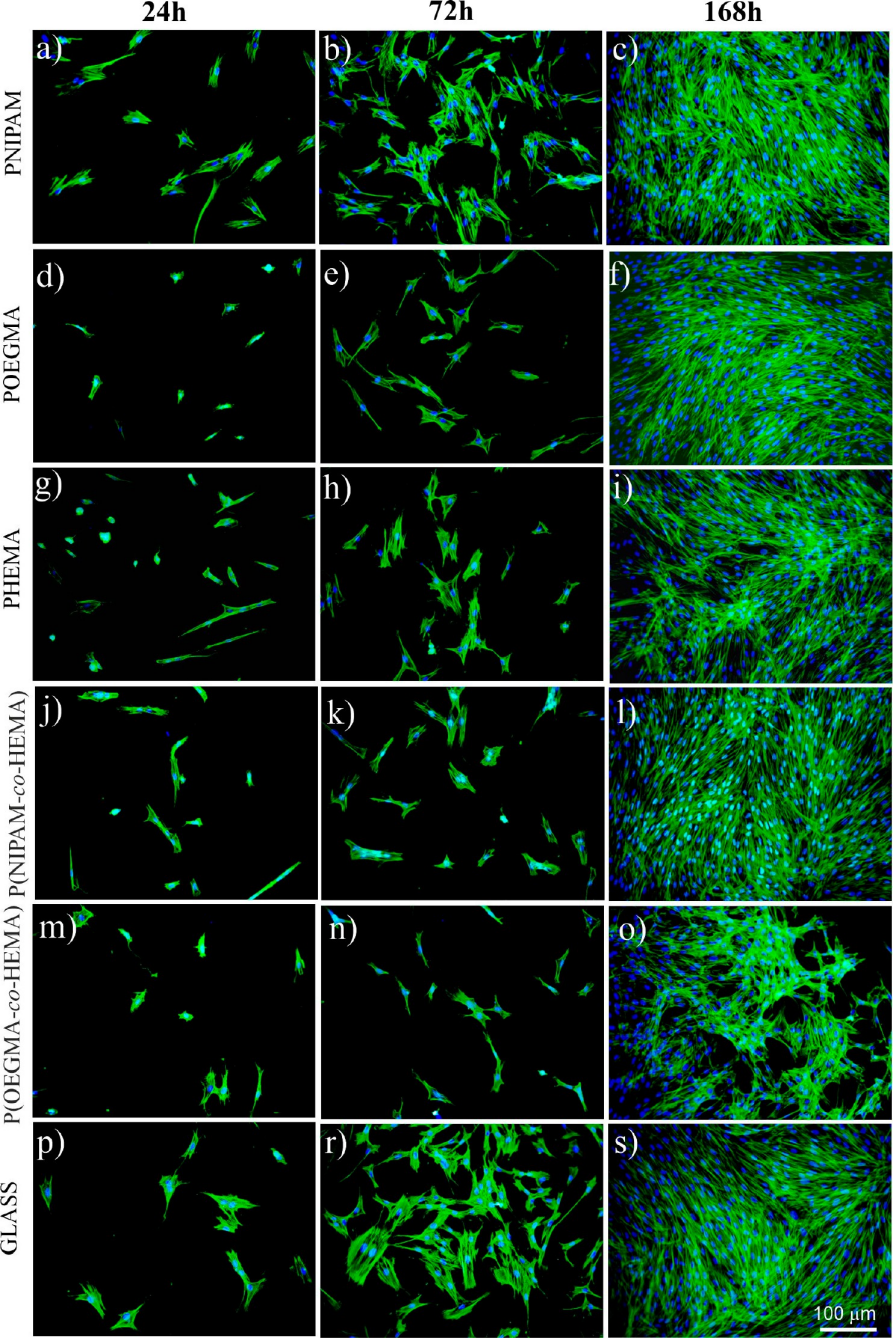
Fluorescence images (cytoskeleton, green; nuclei, blue) of dermal fibroblasts cultured on the fabricated coatings.

The representative fluorescence micrographs showing the cells after 24, 72, and 168 h of culture on all examined polymer coatings and on the control glass sample are presented in Figure 6. After 24 h, rare, distant, and well-spread fibroblasts are visible on the glass substrate (Figure 6p). After 72 h of culture, the number of fibroblasts grows significantly; they are flattened, and their spreading area is large, suggesting a good condition of the cells (Figure 6r). After the longest incubation time, the number of fibroblasts noticeably increases again and forms a confluent monolayer, confirming good culture conditions (Figure 6s). Similar cellular behavior may be observed on the PNIPAM coating (Figure 6a–c), but here, the spreading area is slightly smaller.

In turn, for fibroblasts cultured on POEGMA coating (Figure 6d–f) after 24 h, the number of cells is slightly reduced as compared to PNIPAM, and the spreading area is significantly smaller. This situation changes for longer incubation times, and after 72 h, fibroblasts’ shapes start to resemble the ones observed for PNIPAM coating, but their spreading area remains lower. This rearrangement may be linked with the fact that, over longer periods of time, cells usually develop an extracellular matrix, thus increasing their ability to adhere.^74^ After 168 h of culture, the confluent cell sheet is formed. In the case of the PHEMA brush (Figure 6g–i), the number of cells observed after 24 h of culture is similar to that observed for the POEGMA coating; however, some of them form round shapes, which may suggest not optimal conditions for cellular culture.^13^ Similar results were reported in other research, reporting altered cell shape^75^ and the formation of aggregates of round malignant melanoma cells^76^ for cell culture performed on unmodified PHEMA substrates. After 72 h, the number of cells is slightly reduced as compared to the PNIPAM coating, but the spreading area is comparable to it. After 168 h, cells cover almost all accessible surfaces; however, the formed layer is not completely confluent, and cells tend to aggregate. The fluorescence micrographs recorded for fibroblasts cultured on the P(NIPAM-*co*-HEMA) copolymer brush (Figure 6j–l) resemble the ones recorded for PNIPAM; however, here, both the number of cells and their spreading area are slightly reduced. In contrast, after 24 and 72 h of culture on P(OEGMA-*co*-HEMA) brush, the number of fibroblasts is significantly lower as compared to other coatings, and their spreading area is very small (Figure 6m,n), suggesting an adverse effect of this material on cells. Additionally, after 168 h of culture, cells form aggregates of cells growing on each other (Figure 6o), instead of adhering to the substrate, and this effect is significantly stronger than for the pure PHEMA coating, which strengthens the hypothesis of limited biocompatibility of the P(OEGMA-*co*-HEMA) brush. A similar arrangement of fibroblasts was reported for cells cultured in an adverse environment, where cell–cell interactions are favored over cell–substrate ones.^77−79^

**Figure 7:**
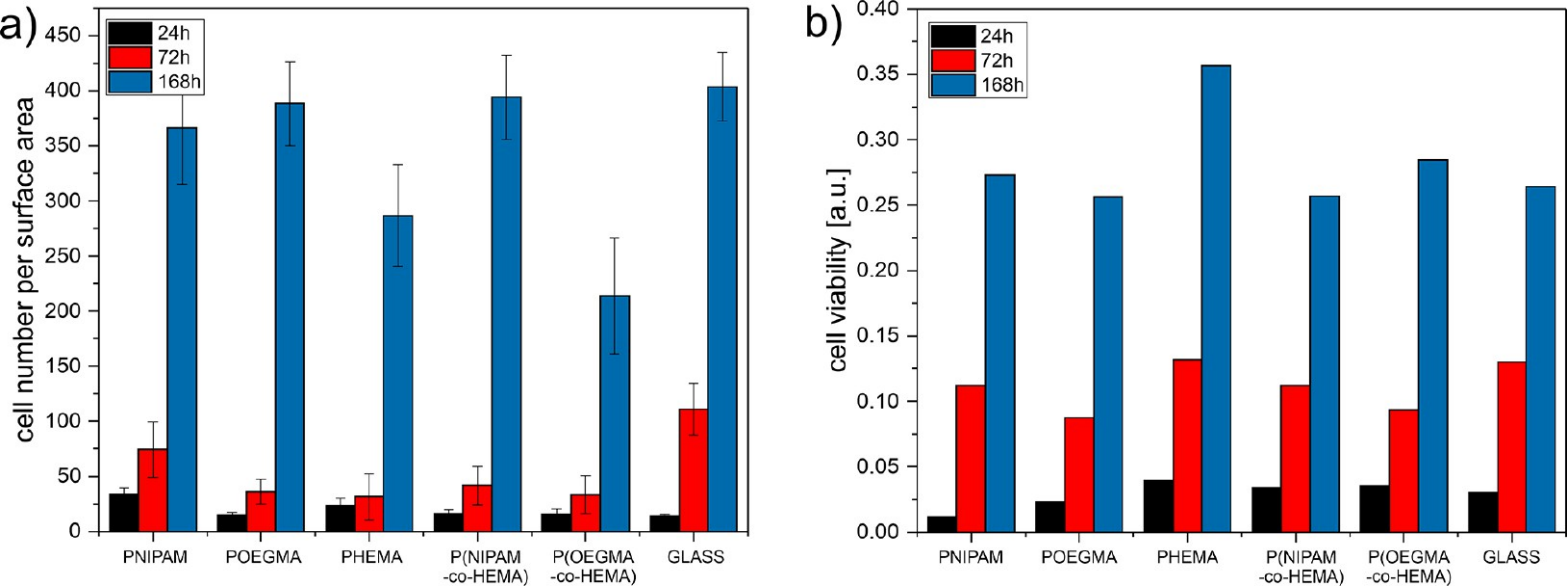
Number (a) and viability (b) of dermal fibroblasts cultured on the fabricated coatings.

The fluorescence micrographs were analyzed quantitatively to determine the cell number per surface area for each fabricated coating (Figure 7a). The obtained results confirm the general observations. For 24 h of culture, the number of cells is highest for PNIPAM and noticeably smaller for all other coatings, and this tendency remains almost the same for 72 h of culture. However, here, the number of cells on the glass substrate grows significantly and becomes the highest. In turn, for the longest incubation time, the number of cells is comparable for the coatings, where the formation of a confluent layer was observed. In turn, the number of cells determined from fluorescence micrographs is reduced for the coatings where the aggregation of cells was observed, significantly more for the P(OEGMA-*co*-HEMA) coating, for which the formation of aggregates was much more effective. However, it should be noted that the results of quantitative analysis in the case of cells growing on each other may be disturbed due to the overlapping of counted cells and the determined values may be underestimated. Therefore, the impact of the coatings on the growth of cells should be verified by using an independent experimental method.

To validate the results obtained using fluorescence microscopy, the viability of the cells was examined using MTT colorimetric tests based on the conversion of MTT into formazan crystals by living cells, which shows mitochondrial function.^80^ The obtained results show that, after 24 h of culture, the viability of cells is highest for the PHEMA coating and lowest for the PNIPAM one (Figure 7b). After 72 h of culture, the viability of cells is comparable for all coatings. Similar results are visible also for 168 h of culture, with the exception of the PHEMA coating, where the viability of dermal fibroblasts is significantly higher than for other coatings. Moreover, the viability of cells cultured on the P(OEGMA-*co*-HEMA) coating is not reduced compared to the coatings where the formation of confluent layers was observed. These results suggest that both the shape and degree of spreading of dermal fibroblasts do not correlate with their viability; thus, the adverse effect of PHEMA and P(OEGMA-*co*-HEMA) coatings postulated based on analysis of fluorescence micrographs is not supported by the MTT results. The observed arrangement of cells leading to the formation of aggregates is not caused by reduced viability of cells on these substrates.

**Figure 8:**
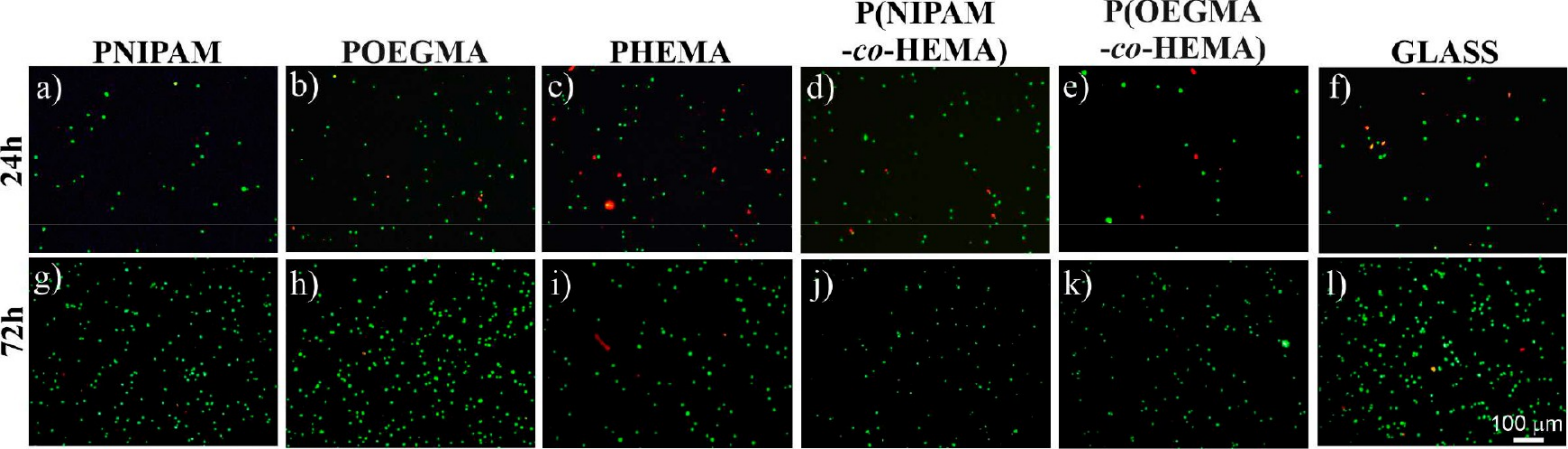
Live (green)/dead (red) staining of dermal fibroblasts cultured on the fabricated coatings.

Additionally, simultaneous fluorescence staining of viable (green) and dead (red) cells was applied to examine the cytotoxicity of the fabricated coatings (Figure 8). Obtained fluorescence micrographs depict that, after 24 h of culture, the majority of cells are viable; however, dead cells are also visible, and their number depends on the coating used as the substrate for cell culture.

**Figure 9:**
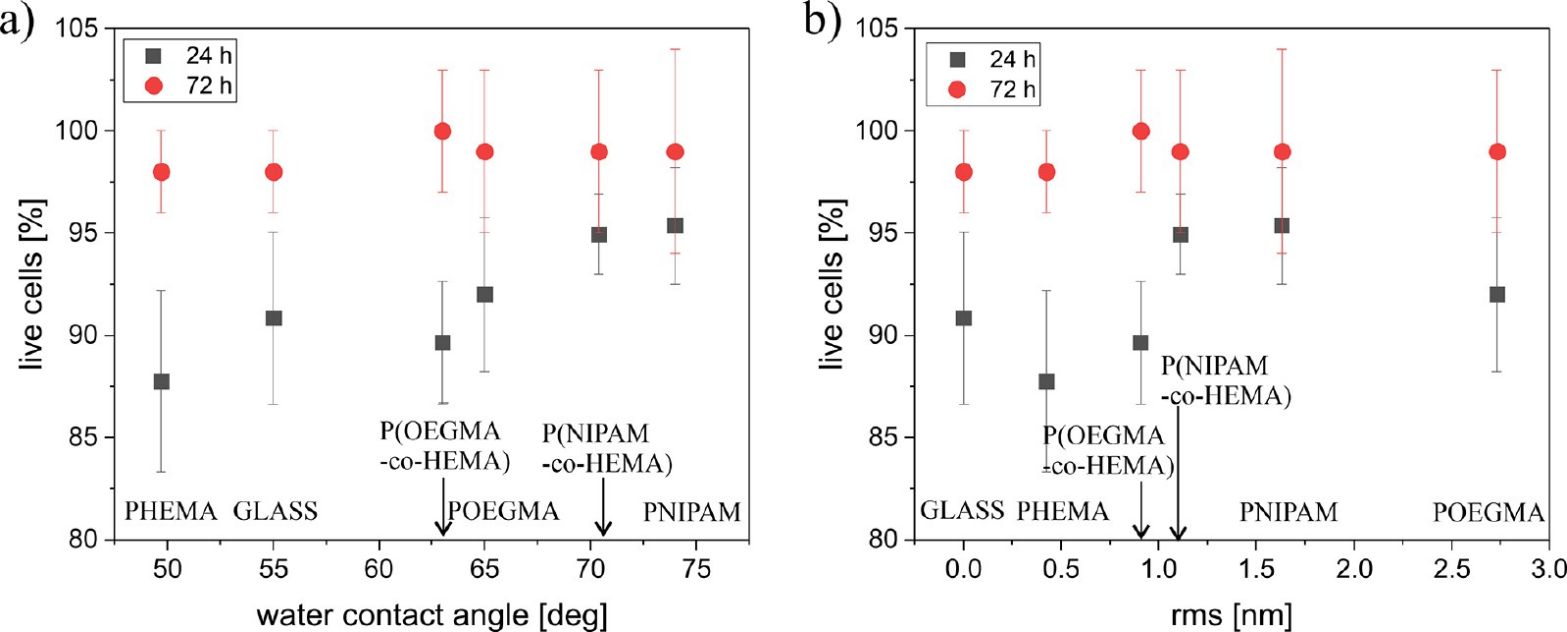
Ratio of viable dermal fibroblasts vs the wettability (a) and RMS roughness (b) of the coating used as a substrate for cell culture.

The number of dead cells is the greatest for PHEMA (Figure 8c), intermediate for POEGMA (Figure 8b) and P(OEGMA-*co*-HEMA) (Figure 8e), and the lowest for PNIPAM (Figure 8a) and P(NIPAM-*co*-HEMA) (Figure 8d) coatings. In turn, after 72 h of culture, almost only viable cells are visible on all coatings. These results indicate that none of the coatings are cytotoxic to the dermal fibroblasts, and the presence of dead cells for the shortest culture time might be more related to the ability of the initial adhesion to the substrates than to their cytotoxicity. To verify this hypothesis, live and dead fluorescence micrographs were analyzed quantitatively to determine the percentage of living cells on each substrate. As the adhesion of cells is mainly determined by surface wettability,^81^ the calculated viability of cells after 24 and 72 h of culture was plotted versus the water contact angle recorded for each substrate at 37 °C (Figure 9).

The obtained results indicate a strong correlation between the cell viability on the given substrate and its wettability for a short culture time (Figure 9a). The more hydrophobic the coating, the greater the number of viable cells observed. Dermal fibroblasts belong to the adhesive cells, which means that they grow and proliferate only when they are adhered to the substrate. Therefore, this observation supports the hypothesis that the differences in viability of cells observed for different coatings might be correlated with the different adhesive potentials of fabricated polymer brushes. However, it should be noted that the changes in wettability are also related to the chemical composition of the polymer and the presence of various chemical groups, which may also affect the cellular adhesion to the coatings. In turn, after 72 h of culture, more than 98% of cells are viable, and no differences may be observed for cells cultured on different coatings. This effect may be linked with the fact that, for longer times, cells usually develop an extracellular matrix, thus increasing their ability to adhere.^74^

**Figure 10:**
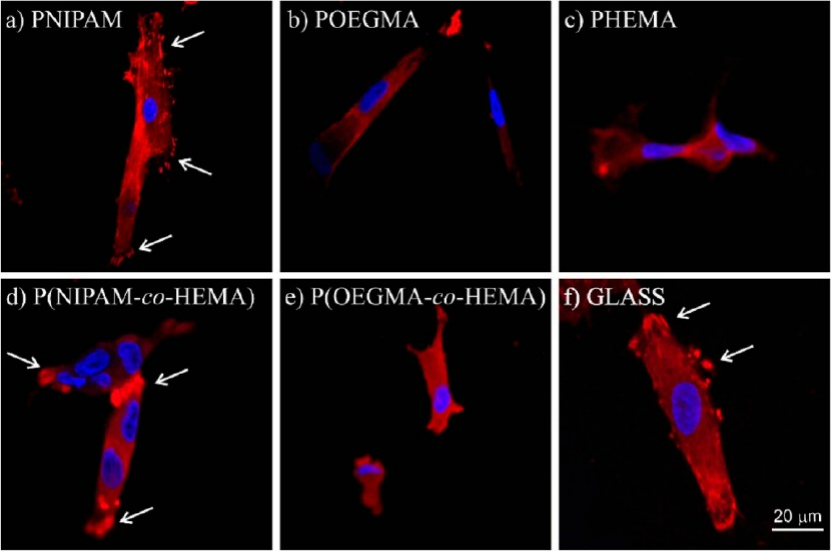
Focal adhesions formed on fabricated polymer brushes and traced by vinculin staining (red).

However, surface properties other than wettability, e.g., may also affect cell behavior. The impact of different structural and topographical cues on nano- and microscales on cellular behavior is widely considered, mainly when developing materials for applications such as medical implants, cell culture systems, or scaffolds for tissue engineering,^82,83,92,84−91^ showing a great variety of cellular responses depending on the specific topographic pattern.^82,93−100^ In addition to the direct impact on whole cell behaviors,^101,102^ effects of nanoscopic scale topography on subcellular mechanisms and the adsorption of extracellular matrix proteins should also be considered.^89,103,104^ In general, increasing the roughness of material surfaces can improve cell adhesion;^89^ however, the impact of topography is cell dependent.^105,106^ The great majority of research addresses the impact of nanotopographies larger than 10 nm, but studies for smaller structures were also performed.^107^ To investigate this issue, cell viability was analyzed as a function of substrate roughness ranging from 0.4 to 3 nm (Figure 9b). Obtained results show that, for short culture times, cells cultured on substrates with RMS values below 1 nm, i.e., glass, PHEMA, and P(OEGMA-*co*-HEMA), show lower viability as compared to more rough coatings. For longer incubation times, i.e., 72 h, no correlation between the coating RMS and cell viability is observed. As increasing roughness of material improves cell adhesion,^89^ the presented analysis suggests that, for short incubation times, cell viability is related to their ability to adhere to examined polymer brushes, which strengthens the conclusions postulated for the impact of the wettability on cell viability (Figure 9a). Cell adhesion is coordinated by many proteins that localize to sites of cell–matrix interaction, so-called “focal adhesions”. One of the best characterized is vinculin, a cytoplasmic actin-binding protein enriched in focal adhesions and adherens junctions.^108−110^ Therefore, to verify the hypothesis that the differences in viability of cells might be related to the different adhesive potentials of fabricated polymer brushes, the formation of focal adhesion at the early adhesion stages was traced by vinculin staining (Figure 10).

Recorded fluorescence micrographs, presenting vinculin (red) and nuclei (blue), show that after 24 h of culture focal adhesions are well developed only for fibroblasts cultured on PNIPAM and P(NIPAM-*co*-HEMA) brushes, while they cannot be noticed on other polymeric coatings, indicating a strongly retarded adhesion process, as compared to the PNIPAM-based ones. These results allow us to relate the viability of cells cultured on polymeric substrates with their adhesive potential, as the highest number of viable cells was observed for PNIPAM and P(NIPAM-*co*-HEMA) coatings, where the formation of focal adhesions is the most effective. In turn, for the glass substrate, other factors, such as elasticity or chemical composition, must be considered to explain reduced cell viability despite the good adhesive properties.

### 3.4 Control of Cell Morphology, Adhesion, and Detachment

Finally, the possibility of using the fabricated P(EGMA-*co*-HEMA) and P(NIPMA-*co*-HEMA) coatings as materials for the CSE platforms was investigated. It is well-known that changes in the surface properties and, as a result, in the morphology of the cells adhered to surfaces determine their further fate, especially detachment. To study this phenomenon, the coating temperature was reduced to 10 °C, i.e., below the transition temperature, and cells were visualized in situ using optical microscopy.

**Figure 11:**
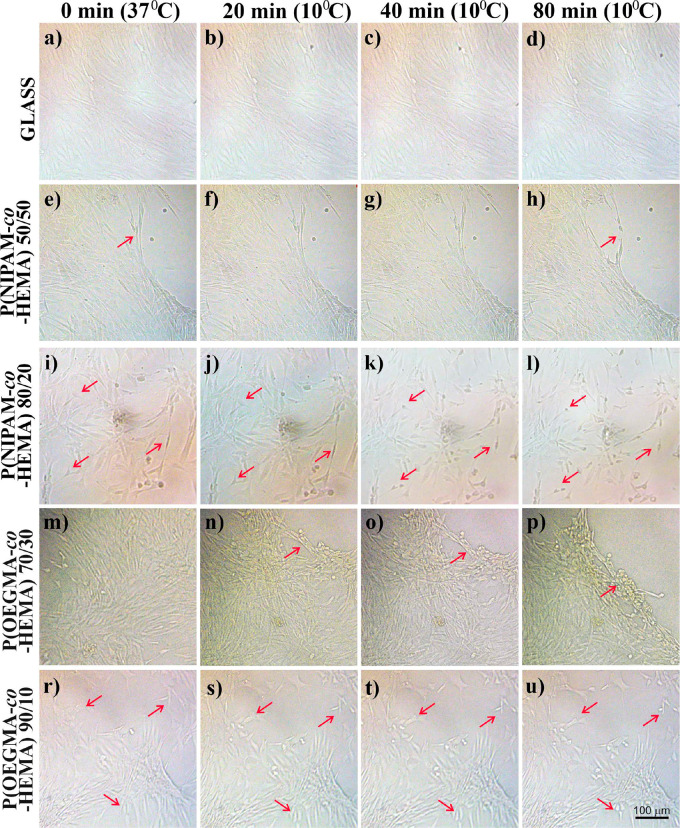
Spontaneous detachment of cells cultured on glass (a–d), P(NIPAM-*co*-HEMA) 50/50 (e–h), P(NIPAM-*co*-HEMA) 80/20 (i–l), P(OEGMA-*co*-HEMA) 70/30 (m–p), and P(OEGMA-*co*-HEMA) 90/10 (r–u) after 0 (a, e, i, m, r), 20 (b, f, j, n, s), 40 (c, g, k, o, t), and 80 (d, h, l, p, u) min incubation at temperature lowered to 10 °C.

The response of the cell sheets to the incubation in temperature lowered to 10 °C for 20, 40, and 80 min (Figure 11) depends strongly on the material used as the cell culture substrate. For glass (Figure 11a–d), no effect of cooling is visible, in either the cell number or their morphology. Similarly, for the P(NIPAM-*co*-HEMA) 50/50 coating (Figure 11e–h), which does not present any temperature-driven transition (see Figure 4b), the flat layer of cells adhered to the substrate is visible even for the longest time of cooling. In turn, for the P(NIPAM-*co*-HEMA) 80/20 coating, which undergoes the LCST driven transition at ∼14 °C, the slight influence of lowered temperature might be observed: the cells significantly change their morphology from elongated, flattened structures into round ones, indicating decreasing adhesion with increasing time of cooling. Most probably, this effect could lead to the detachment of cells for longer incubation times or lowering the temperature of incubation, which in the present study is only slightly lower than the transition temperature. These results are in agreement with the literature data, reporting very slow detachment of cell sheets from surfaces of TCPS grafted with PNIPAM (∼75 min), occurring gradually from the periphery of the sheet toward the interior.^111^ A similar situation occurs for the P(OEGMA-*co*-HEMA) 90/10 coating (Figure 11r–u), which also exhibits LCST transition; however, here, the transition temperature is noticeably higher (∼20 °C); thus, the response of cells is more evident. Fibroblasts change their morphology and start to detach from the surface already after 20 min of cooling. However, cell detachment is the most pronounced for the P(OEGMA-*co*-HEMA) 70/30 coating, for which UCST-driven transition was recorded, with transition temperature at ∼27 °C. Already after 20 min of incubation in lowered temperature, a large part of the cell sheet detaches, and this process continues with increasing cooling time, resulting in the complete detachment of approximately half of the observed cell sheet after 80 min (Figure 11p). Spontaneous cell detachment was reported for UCST-based materials composed of poly(*N*-acryloyl glycinamide-*co*-*N*-phenylacrylamide) copolymers, which were used as substrates for NIH-3T3 adhering to the brushes at 30 °C, below the UCST transition, and releasing it at 37 °C.^63^ In the case of the P(OEGMA-*co*-HEMA) 70/30 coating examined in this paper, the opposite effect is observed, and cells adhere and grow at 37 °C (above the UCST) and are released from the surface after the temperature is lowered to 10 °C, which is far below the transition temperature. Cell adhesion depends strongly on the wettability of the surface.^14,15,43−47^ However, this dependence is not linear: the research performed for various cells (like Chinese hamster ovary cells, fibroblasts, endothelial cells, and rat pheochromocytoma) showed maximal adhesiveness, and the best growing conditions for cells are obtained for moderately hydrophilic substrates with a contact angle of around 55°.^112−114^ Therefore, the observed detachment of cells from the P(OEGMA-*co*-HEMA) 70/30 coating may be related to the change in surface wettability resulting in the rapid deterioration of cell culture conditions, optimal for 37 °C (above the UCST).

**Figure 12:**
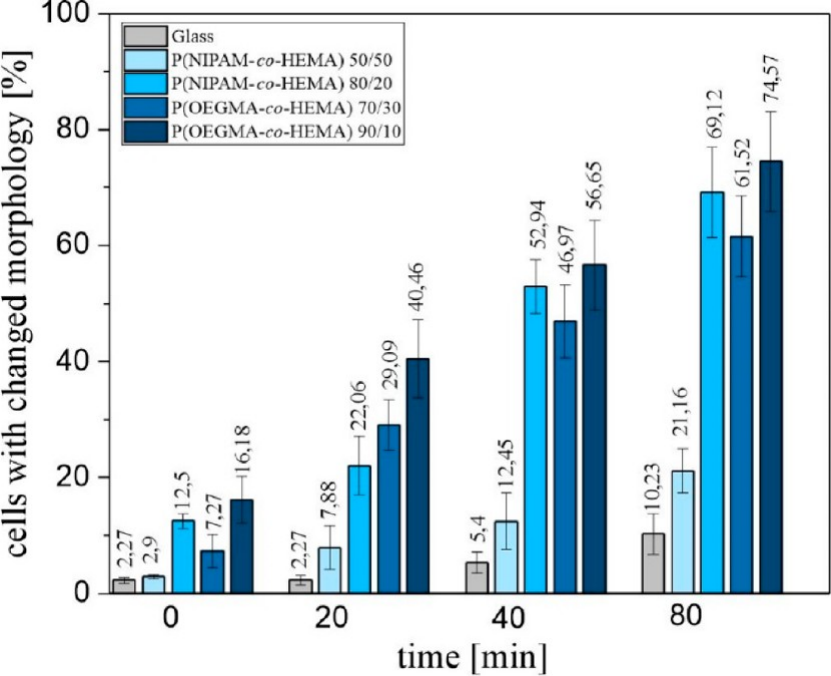
Fraction of cells with changed morphology cultured on glass, P(NIPAM-*co*-HEMA) 50/50, P(NIPAM-*co*-HEMA) 80/20, P(OEGMA-*co*-HEMA) 70/30, and P(OEGMA-*co*-HEMA) 90/10 after 0, 20, 40, and 80 min of incubation at a temperature lowered to 10 °C.

To analyze quantitatively the impact of cooling on cells cultured on different substrates, the fraction of cells with the morphology affected by a decreased temperature was calculated (Figure 12).

Prior to the lowering of temperature, the number of cells with the morphology affected by substrate properties were calculated (Figure 12, *t* = 0), showing that, for glass and P(NIPAM-*co*-HEMA) 50/50 coating, almost all cells are flattened, whereas for other coatings, also round-shaped cells are visible, and their fraction equals 7% for P(OEGMA-*co*-HEMA) 70/30, 12.5% for P(NIPAM-*co*-HEMA) 80/20, and 16% for P(OEGMA-*co*-HEMA) 90/10. Increasing the time of incubation to 10 °C leads to the linear growth of the number of modified cells for all substrates. This effect is weakest for glass, where the fraction of modified cells does not exceed 10%, even for the longest incubation time. Similarly, for the P(NIPAM-*co*-HEMA) 50/50 coating, which does not show any thermoresponsiveness, the number of cells with changed morphology is relatively low, reaching approximately 20% of all cells after 80 min of cooling. In turn, for the copolymer coatings exhibiting a temperature transition, either LCST or UCST driven, the impact of temperature lowering is significant. For the shortest recorded cooling time, i.e., 20 min, there are noticeable differences in the fraction of modified cells observed on each coating, which equals 20% for P(NIPAM-*co*-HEMA) 80/20, almost 30% for P(OEGMA-*co*-HEMA) 70/30, and 40% for P(OEGMA-*co*-HEMA) 90/10. With increasing time of cooling, the difference in the number of transformed cells cultured on coatings with LCST-driven transition, i.e., P(NIPAM-*co*-HEMA) 80/20 and P(OEGMA-*co*-HEMA) 90/10, vanishes, approaching 55% and 70%, for incubation for 40 and 80 min, respectively. In contrast, the fraction of cells with modified morphology observed on P(OEGMA-*co*-HEMA) 70/30, with UCST-based transition, is slightly lower and equals 47% and 61% after cooling for 40 and 80 min, respectively.

The changes in cell morphology and their tendency to release the surface of thermoresponsive coatings confirm the predicted ability of coatings for noninvasive, enzyme-free spontaneous detachment of cells induced by lowered temperature. Surprisingly, in addition to materials with LCST-driven transition, this effect occurs also for UCST-driven materials with properly adjusted wettability. Moreover, the presented results suggest different mechanisms of action depending on the type of transition. For coatings with UCST, the lowering of the temperature results in an effective detachment of cells, whereas for polymer brushes with LCST, the modification of cell morphology dominates. However, this hypothesis requires further detailed studies.

## 4 Conclusions

In the framework of this Article, five polymer brush coatings composed of POEGMA, PNIPAM, PHEMA, P(OEGMA-*co*-HEMA), and P(NIPAM-*co*-HEMA), which could potentially serve as materials for CSE platforms, were fabricated and characterized. The performed analysis of the physicochemical properties of the coatings confirmed the successful fabrication of the coatings, with similar thickness and roughness. In turn, the examination of the response of the coatings to the temperature stimulus revealed that the thermoresponsiveness of copolymer brushes depends strongly on their composition and may be preserved, vanish, or change the mechanism from LCST to UCST driven for different molecular ratios of monomers. To examine the cytotoxicity of coatings, they were used as substrates for the culture of dermal fibroblasts, whose growth and viability were analyzed quantitatively, showing that none of the coatings are cytotoxic to examined cells. However, the shape and arrangement of cells depend significantly on the composition of the coating. For PHEMA and P(OEGMA-*co*-HEMA) coatings, cells form agglomerates, growing preferentially on each other and not on the polymer coating. Moreover, the viability of the cells was related to the wettability and roughness of the coatings, which determined the initial adhesion of cells. Finally, the noninvasive, enzyme−free spontaneous detachment of cells as well as modification of cell morphology, caused by changes in the properties of the fabricated copolymer coatings induced by lowered temperature were presented.

The performed experiments revealed the numerous advantages of the proposed coatings. First, they present different types of thermoresponsiveness, which makes them very interesting objects for basic studies aimed at understanding the molecular mechanisms of thermal response. Second, the transition temperature of the coatings may be easily shifted by the modification of their composition. Third, the coatings are not cytotoxic to human cells, which are able to spontaneously detach from the surface when the temperature is lowered. These properties make fabricated coatings promising candidates for CSE platforms, which nowadays are based mainly on LCST-type materials and hardly on UCST-based ones and for which applications are sparsely explored. Moreover, they have a great capacity for further modifications, as ensured by the HEMA units, and the aim should be to enhance their application potential by increasing the cell adhesion and reducing the time of detachment. Finally, different mechanisms of cell–substrate interactions were concluded for coatings with UCST and LCST, presenting an effective detachment of cells and a strong modification of the cell morphology, respectively. However, this hypothesis requires further detailed studies.

## Supporting Information Available

Topography (Figure S1) and thickness and RMS (Table S1) of the representative coatings; type and temperature of transition of the coatings (Table S2); experimental details of atomic force microscopy and profilometry measurements (PDF)
