# Supplementary material for: Thermoresponsive Smart Copolymer Coatings Based on P(NIPAM-co-HEMA) and P(OEGMA-co-HEMA) Brushes for Regenerative Medicine
Source: ACS Biomater Sci Eng. 2023 Oct 24;9(11):6256–72. doi: 10.1021/acsbiomaterials.3c00917 (PMC10646826; doi:10.1021/acsbiomaterials.3c00917)
Supplement: Supplementary file 1 — ab3c00917_si_001.pdf [file ab3c00917_si_001.pdf]

**Supporting Information**

# Thermoresponsive Smart Copolymer Coatings Based on P(NIPAM-*co*-HEMA) and P(OEGMA-*co*-HEMA) Brushes for Regenerative Medicine

**Svitlana Tymetska,[1,2,#] Yana Shymborska, [1,2,3,#] Yurij Stetsyshyn,[3] Andrzej Budkowski, [2] Andrzej Bernasik,[4] Kamil Awsiuk, [2] Volodymyr Donchak, [3] Joanna Raczkowska[2*]**

[1]Jagiellonian University, Doctoral School of Exact and Natural Sciences, Łojasiewicza 11, 30-348 Kraków, Poland

[2]Jagiellonian University, Faculty of Physics, Astronomy and Applied Computer Science, Smoluchowski Institute of Physics, Łojasiewicza 11, 30-348 Kraków, Poland

[3]Lviv Polytechnic National University, St. George's Square 2, 79013 Lviv, Ukraine

[4]Faculty of Physics and Applied Computer Science, AGH - University of Science and Technology, al. Mickiewicza 30, 30-049 Kraków, Poland

[#]Both authors contributed equally to this manuscript

The topography of the coatings was recorded using atomic force microscopy (AFM) depicting similar, island-like structures for all coatings.

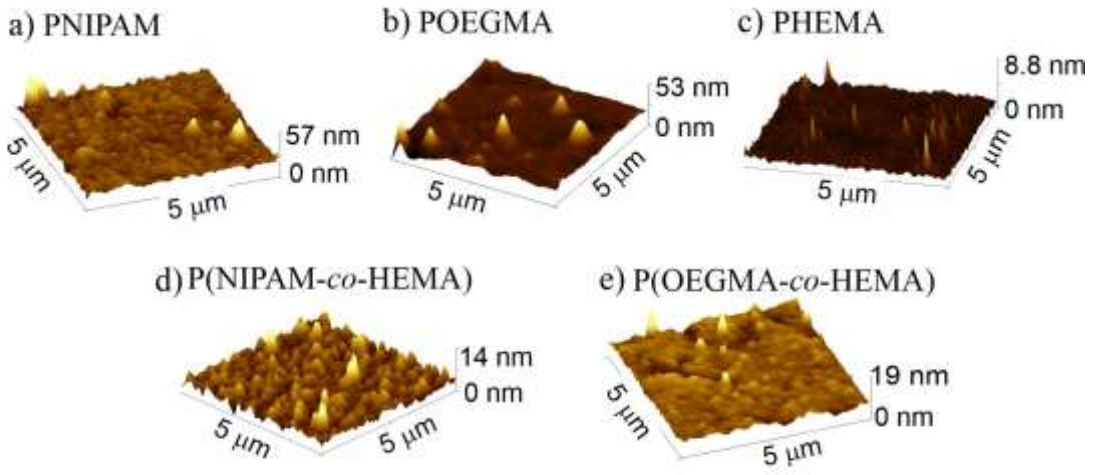


Fig. S1 Topography of the representative coatings, recorded using AFM at room temperature.

However, the numerical analysis of recorded topographies by means of root-mean-square (RMS) roughness analysis (Table S1) indicates that PNIPAM and POEGMA coatings are quite rough (~ 2.5 nm), PHEMA coating is relatively smooth, with RMS value less than half of nanometer whereas the copolymer brushes have intermediate roughness of about 1-1.5 nm.

**Table S1.** Thickness and RMS of the coatings recorded at room temperature

| Coating | RMS [nm] | Thickness [nm] |
|---|---|---|
| PNIPAM | 2.57729 | 42.17 |
| POEGMA | 2.73273 | 46.23 |
| PHEMA | 0.47767 | 44.22 |
| P(NIPAM-*co*-HEMA) | 1.45332 | 43.86 |
| P(OEGMA-*co*-HEMA) | 1.08503 | 45.81 |

Additionally, the thickness of coatings was determined using profilometer, differs only slightly between the coatings and equals to 42 – 46 nm (Table S1).

Thermoresponsiveness of the coatings was determined with water contact angle measurements (CA) as a function of temperature. Then, the transition temperature was determined from the Boltzmann fit to the experimental points

**Table S2.** Thermoresponsiveness of the coatings

| **molar composition** | **P(OEGMA-*co*-HEMA)** | | **P(NIPAM-*co*-HEMA)** | |
|---|---|---|---|---|
| | **transition type** | **transition temperature [$^0$C]** | **transition type** | **transition temperature [$^0$C]** |
| **0/100 (PHEMA)** | **no transition** | **-** | **no transition** | **-** |
| 50/50 | **no transition** | - | **no transition** | - |
| 70/30 | UCST | 26.9 ± 0.5 | LCST | 11.1 ± 1.5 |
| 80/20 | UCST | 19.9 ± 1.5 | LCST | 14.8 ± 2.1 |
| 90/10 | LCST | 19.6 ± 0.8 | LCST | 18.5 ± 1.1 |
| 100/0 | LCST | 16.8 ± 0.3 | LCST | 29.2 ± 0.5 |

**Atomic force microscopy.** Topographic images were recorded on randomly chosen regions of the sample surface. Measurements were carried out in air using the commercially available Agilent 5500 system (Keysight) working in non-contact mode with non-coated super sharp silicon probes. For every coating at least three images were analyzed using provided with the AFM apparatus.

**Profilometry.** To examine thickness of fabricated coatings, they were scratched and the scratch profiles were recorded using a Dektak XT (Bruker, Germany) profilometer, equipped with a 12.5 µm radius stylus. For each sample, at least three profiles were collected in the standard hills and valleys module, to determine the average thickness of the coatings.
